# Early inflammatory profiles predict maximal disease severity in COVID-19: An unsupervised cluster analysis

**DOI:** 10.1016/j.heliyon.2024.e34694

**Published:** 2024-07-23

**Authors:** Grace Kenny, Gurvin Saini, Colette Marie Gaillard, Riya Negi, Dana Alalwan, Alejandro Garcia Leon, Kathleen McCann, Willard Tinago, Christine Kelly, Aoife G. Cotter, Eoghan de Barra, Mary Horgan, Obada Yousif, Virginie Gautier, Alan Landay, Danny McAuley, Eoin R. Feeney, Cecilia O'Kane, Patrick WG. Mallon

**Affiliations:** a Centre for Experimental Pathogen Host Research, University College Dublin, Dublin, Ireland; b Department of Infectious Diseases, St Vincent's University Hospital, Dublin, Ireland; c Department of Infectious Diseases, Mater Misericordiae University Hospital, Dublin, Ireland; d Department of International Health and Tropical Medicine, Royal College of Surgeons in Ireland, Dublin, Ireland; e Department of Infectious Diseases, Cork University Hospital, Wilton, Cork, Ireland; f Department of Endocrinology, Wexford General Hospital, Wexford, Ireland; g Department of Internal Medicine, Rush University, Chicago, IL, USA; h Queen's University Belfast, Belfast, United Kingdom

**Keywords:** Severe acute coronavirus 2, Immune phenotypes, Biomarkers, Principal component analysis

## Abstract

**Background:**

The inflammatory changes that underlie the heterogeneous presentations of COVID-19 remain incompletely understood. In this study we aimed to identify inflammatory profiles that precede the development of severe COVID-19, that could serve as targets for optimised delivery of immunomodulatory therapies and provide insights for the development of new therapies.

**Methods:**

We included individuals sampled <10 days from COVID-19 symptom onset, recruited from both inpatient and outpatient settings. We measured 61 biomarkers in plasma, including markers of innate immune and T cell activation, coagulation, tissue repair and lung injury. We used principal component analysis and hierarchical clustering to derive biomarker clusters, and ordinal logistic regression to explore associations between cluster membership and maximal disease severity, adjusting for known risk factors for severe COVID-19.

**Results:**

In 312 individuals, median (IQR) 7 (4–9) days from symptom onset, we found four clusters. Cluster 1 was characterised by low overall inflammation, cluster 2 was characterised by higher levels of growth factors and markers of endothelial activation (EGF, VEGF, PDGF, TGFα, PAI-1 and *p*-selectin). Cluster 3 and 4 both had higher overall inflammation. Cluster 4 had the highest levels of most markers including markers of innate immune activation (IL6, procalcitonin, CRP, TNFα), and coagulation (D-dimer, TPO), in contrast cluster 3 had the highest levels of alveolar epithelial injury markers (RAGE, ST2), but relative downregulation of growth factors and endothelial activation markers, suggesting a dysfunctional inflammatory pattern. In unadjusted and adjusted analysis, compared to cluster 1, cluster 3 had the highest odds of progressing to more severe disease (unadjusted OR (95%CI) 9.02 (4.53–17.96), adjusted OR (95%CI) 6.02 (2.70–13.39)).

**Conclusion:**

Early inflammatory profiles predicted subsequent maximal disease severity independent of risk factors for severe COVID-19. A cluster with downregulation of growth factors and endothelial activation markers, and early evidence of alveolar epithelial injury, had the highest risk of severe COVID-19.

## 1 Introduction

The past two decades have witnessed a number of severe pandemic viral respiratory infections, including COVID-19, Middle East Respiratory Syndrome (MERS), influenza H1N1 and Severe Acute Respiratory Syndrome (SARS), and a growing body of literature suggests the risk of future pandemics is increasing [1,2]. The COVID-19 pandemic offered a unique opportunity to advance our understanding of the host immune response to a novel respiratory virus with severe disease outcomes. This could help improve therapeutic strategies for future novel pathogens. A minority of those infected with SARS-CoV-2 require hospitalisation for supplemental oxygen therapy, or higher levels of respiratory support [3]. COVID-19 typically progresses through multiple phases in those who experience severe disease, a viral symptom phase, an early inflammatory phase (day 7 [[4], [5]]), and a late inflammatory phase (day 10.5-12 [[4], [5]]), which may be complicated by cytokine storm, coagulopathy and secondary infection [6,7]. The efficacy of therapies for COVID-19 are dependent on administration of the correct therapy at the appropriate time in the disease course. For example, corticosteroids are beneficial in hospitalised individuals requiring oxygen therapy but may be harmful in those not requiring respiratory support [8]. Similarly, the IL-6 inhibitor tocilizumab, which is recommended for those who have rapidly increasing oxygen requirements despite corticosteroids [9], has had conflicting results in randomised controlled trials. In some trials, these differences were attributed to administration too late in the disease course [10].

Early identification of individuals likely to deteriorate can be challenging. Broad factors such as age and obesity have been identified as risk factors for more severe disease [11,12], but many of these individuals will experience a benign course. Diagnostic tests to identify those who are progressing to more severe disease who would derive the greatest benefit from directed immunotherapy are not readily available. While multiple studies have associated individual inflammatory biomarkers with subsequent severe disease [[13], [14], [15], [16]], such analyses fail to adequately depict the heterogeneity in host inflammatory response. Failure to account for pathophysiologic heterogeneity has limited treatment advances in other conditions featuring dysregulated immune responses, such as sepsis and acute respiratory distress syndrome (ARDS). Recently, identification of immunophenotypes that predict outcome and treatment response has offered promise in these conditions [[17], [18], [19]]. Phenotypes that predict response to steroids have been demonstrated in COVID related ARDS requiring mechanical ventilation [20], but detailed phenotyping of the cytokine changes that precede the development of severe disease, the optimal window for intervention, are lacking.

To address this research gap, in this study we aimed to characterise inflammatory phenotypes in a large cohort of individuals with early COVID-19. We identify phenotypes that are at greater risk of progression to severe COVID-19, that could serve as targets for specific immunomodulatory treatment, or provide insights for the development of new therapies.

## 2 Methods

### 2.1 Study participants

The All Ireland Infectious Disease (AIID) cohort study is a prospective, multi-centre, observational cohort study that recruits individuals attending clinical services for issues relating to infectious diseases from nine hospitals in Ireland, as previously described [[21], [22], [23]]. Participants provide written, informed consent for the collection of demographic and clinical data and the collection of blood samples for biobanking, from which ethylenediaminetetraacetic acid (EDTA) derived plasma is stored at −80 °C until analysis. For this study, we included individuals with a biobanked sample available ≤10 days from the date of acute symptom onset, prior to the typical time of progression to severe disease [5,24]. Symptom onset was determined based on participant self-report. We selected a time period prior to the widespread rollout of COVID-19 vaccination [25] in order to examine host inflammatory profiles in a non-immune population. The AIID Study and these analyses were approved by the National Research Ethics Committee approval number 20-NREC-COV-056.

#### 2.1.1 Cytokine analysis

We measured 61 biomarkers encompassing markers of innate immune activation [hsCRP, IL-6, IL-8, IL-15, IL-17 A, IL-18, procalcitonin, sCD163, CCL2 (MCP1), CCL3 (MIP-1⍺), CXCL10, GM-CSF, TNF-R1, GDF-15], Th1 activation [IL-1β, IL-2, 2 L-12p70, TNF⍺], Th2 activation [IL-4, IL-5, IL-13, IL-33, TSLP, interferons, (IFN-⍺2, IFN-β, IFN-ɣ, IFN-λ1 (IL-29), IFN-λ2 (IL28A), IFN-λ3 (IL-28 B], endothelial activation [ICAM-1, sVCAM-1, e-selectin, *p*-selectin], tissue repair [VEGF, EGF, PDGF-AA, PDGF-AB, TGF⍺], coagulation [vWF, D-dimer, TPO, CD40L, plasminogen activator inhibitor-1 (PA-1)], gut barrier integrity [zonulin, beta-D-glucan, LBP, I-FABP, sCD14], immune regulation [IL1RA, PD-L1, IL-10, TNF-R2], adipokines [adiponectin, leptin, FABP4, resistin], axonal injury [S100B] and alveolar epithelial injury [ST2, SPD, RAGE]. Biomarkers were measured using either customised MILLIPLEX multiplex assays using Luminex Technology (Merck, Darmstadt, Germany) or customised multiplex V-PLEX panels using Mesoscale Diagnostics (MSD) electrochemiluminescence technology (Mesoscale diagnostics LLC, Rockville, Maryland, USA). I-FABP (MSD and Luminex), zonulin, beta-D-glucan (Assay Genie, Dublin, Ireland), GDF-15, SPD, ST2, and RAGE (R&D Systems, Minneapolis, Minnesota, USA) were run in singleplex. Biomarker panels are outlined in Supplementary Table 1. All samples were run in duplicate along with control samples and standard curves on each plate, according to the manufacturer's instructions.

### 2.2 Statistical analysis

Continuous variables were summarised with median and interquartile range (IQR) and compared using the Kruskal Wallis or Wilcoxon rank sum test. Categorical variables were summarised by number and percent and compared using the chi square or Fisher's exact test. We adopted methodology previously used to identify inflammatory phenotypes in people living with HIV for this study [26,27]. First, to reduce the dimensionality of the dataset, we used principal component analysis (PCA) on the 61 biomarkers. PCA is a statistical technique that aims to explain the covariance structure of a set of variables through a few linear combinations of the original variables. By retaining only the components that explain most of the variance of the dataset, PCA serves as a dimension reduction technique. Prior to PCA, biomarkers were log-transformed and scaled. Missing biomarker measurements were imputed using the regularised PCA iterative algorithm. The number of principal components (PCs) retained were those that contained a greater proportion of variation than those obtained by the 0.95 quantile of variation of data tables simulated on the basis of a normal distribution. Next, we performed cluster analysis to identify groups of similar observations. Hierarchical clustering was performed on the retained PCs, using Ward's minimum variance linkage and squared Euclidean distance as a distance measure. The number of clusters was chosen by calculating the within cluster sum of squares (inertia) for each solution and cutting the dendrogram at the point with the greatest loss of inertia. K means consolidation was performed following hierarchical clustering, which minimises the within cluster sum of squares for each cluster.

We used univariable and multivariable ordinal logistic regression with maximal World Health Organisation disease severity [28] as the dependent variable, to explore association with cluster membership. For the ordinal logistic regression, we collapsed disease severity to three levels, mild, moderate and severe, with severe and critical being considered in a single severe category. Proportional odds assumption was evaluated using the Brant test [29]. For the multivariable models, we included the following factors which are known to be associated with an increased risk of COVID-19; age, sex, ethnicity, smoking history, diabetes, hypertension and immunosuppression [30,31]. We used multiple imputation with predictive mean matching to handle missing data for the ordinal logistic regression model [32]. Data was missing for 13 % of BMI and <1 % of other variables. All analysis was carried out using R version 4.3.0.

## 3 Results

Between March 2020 and April 2021, 780 individuals with confirmed COVID-19 and symptom onset data provided blood for analysis, of these, 334 were ≤10 days from symptom onset, and 312 had sufficient plasma for analysis. Wild type and Alpha variant SARS-CoV-2 were the dominant variants during this time period [33]. Participant characteristics are shown in Table 1. Median (IQR) age was 62 (48–77) years, 54 % were male, and median (IQR) days from symptom onset was 7 (4–9) days. Maximal disease severity was mild for 154 (49 %) participants (including 47 (15 %) outpatients), moderate for 66 (21 %) and severe for 92 (29 %). 36 (11 %) of the cohort subsequently died as a result of COVID-19.

**Table 1:** Participant characteristics overall and by cohort.

	Whole cohort n = 312	Cluster 1 Less inflamed n = 81	Cluster 2 Tissue repair n = 93	Cluster 3 Tissue injury n = 77	Cluster 4 Systemic inflammation n = 61	P value
**Age (years) (median (IQR))**	62 (48–77)	37 (29–56)	59 (52–71)	73 (60–83)	72 (62–81)	<0.001
**Male sex**	170 (54 %)	35 (43 %)	55 (59 %)	48 (62 %)	32 (52 %)	0.073
**Non Caucasian ethnicity**	63 (20 %)	11 (14 %)	19 (21 %)	14 (18 %)	14 (23 %)	0.5
**BMI (kg/m**^**2**^**) (median (IQR))**	27.56 (24–32)	25 (23–28)	30 (25–32)	27 (24–30)	30 (26–33)	0.001
**Ever smoked**	99 (32 %)	15 (19 %)	30 (32 %)	32 (42 %)	22 (36 %)	0.015
**Hypertension**	133 (42 %)	11 (14 %)	44 (48 %)	44 (57 %)	34 (56 %)	<0.001
**Diabetes**	46 (15 %)	4 (4.9 %)	12 (13 %)	13 (17 %)	17 (28 %)	0.002
**Immunosuppressed**	13 (4 %)	1 (1.2 %)	0 (0 %)	8 (10 %)	4 (6.6 %)	0.0009
WHO Disease severity* Mild **Moderate** **Severe**	154 (49 %) 66 (21 %) 92 (29 %)	64 (79 %) 10 (12 %) 7 (8.6 %)	40 (43 %) 28 (30 %) 25 (27 %)	23 (30 %) 18 (23 %) 36 (47 %)	27 (44 %) 10 (16 %) 24 (39 %)	<0.001
**Days from symptom onset (median (IQR))**	7 (4–9)	6 (4–8)	8 (5–9)	6 (4–8)	6 (3–8)	0.003
**Death**	36 (11 %)	3 (4 %)	3 (3 %)	19 (25 %)	11 (18 %)	<0.001

Legend: Data are number (%) unless otherwise specified. Categorical variables were compared with chi square test and continuous with Kruskal-Wallis test. BMI – body mass index × World Health Organisation disease severity collapsed into three categories for analysis, with severe and critical categories combined. BMI – Body Mass Index.

### 3.1 Characteristics of inflammatory phenotypes

**Fig. 1:**
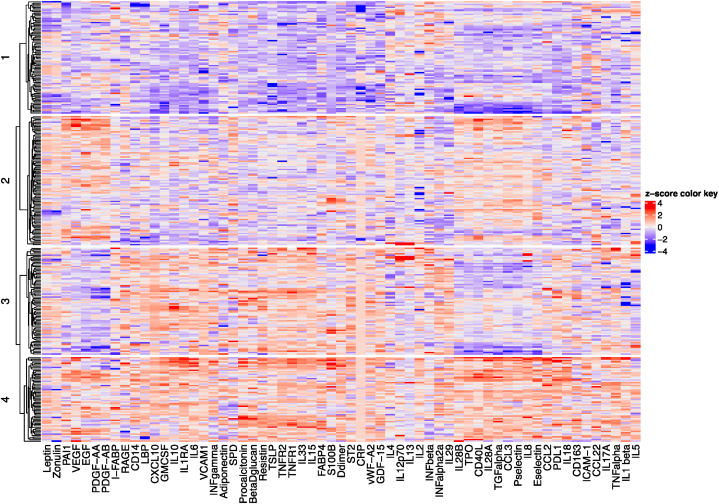
Heatmap demonstrating the inflammatory clusters.

PCA on the 61 biomarkers resulted in eight PCs being retained, accounting for 59.78 % of the total variance. Scree plot and hierarchical clustering dendrogram are shown in Supplementary Figs. 1 and 2. PCA and hierarchical clustering revealed four distinct clusters (Fig. 1). Clinical characteristics of the clusters are shown in Table 1.

The first cluster had 81 (26 %) participants, and was sampled a median (IQR) of 6 (4–8) days from symptom onset. This cluster was characterised by overall low levels of inflammation, with markers across all pathways lower in this cluster than in the other three clusters. This cluster was the youngest (median (IQR) age 37 (29–56) years, p < 0.001), had the lowest BMI (median (IQR) 25 (23–28) kg/m^2^ p = 0.001), and participants in this cluster had the lowest prevalence of comorbidities such as hypertension and diabetes (14 % (p < 0.001) and 4.9 % (p = 0.002) respectively). This cluster also had the lowest proportion of male participants (43 %), although sex differences between clusters did not meet statistical significance (p = 0.073). In line with the low prevalence of risk factors and less inflamed pattern, this cluster had the highest proportion of individuals with mild maximal disease severity (79 %, p < 0.001).

The second cluster (n = 93 (30 %)), was the latest in the disease course, with a median (IQR) of 8 (5–9) days from symptom onset (p = 0.003). This cluster, which we termed a “tissue repair” cluster, had an upregulation of growth factors. In particular, EGF, PDGF-AA and PDGF-AB and alveolar epithelial proliferation marker SPD were highest in this cluster, although VEGF and TGF⍺ were also upregulated compared to clusters 1 and 3 (all p < 0.001). IL13, which has immunoregulatory functions by inhibiting the production of inflammatory cytokines from macrophages [34] and contributes to proliferation of airway epithelial cells [35] and PA-1, which regulates cellular migration and tissue remodelling [36] were also highest in this cluster (p = 0.011 and p < 0.001, respectively). The markers of endothelial activation, *E*-selectin and *P*-selectin, platelet activation TPO and CD40L, as well as type 3 interferons IL28A and IL28B, were all upregulated relative to clusters 1 and 3 (all p < 0.001). Compared to the less inflamed cluster 1, cluster 2 was older (median (IQR) age 59 (52–71) years p < 0.001), had a higher BMI (median (IQR) 30 (25–32) kg/m^2^ p = 0.01); and had a higher proportion of individuals with hypertension and diabetes (48 % (p < 0.001) and 13 % (p = 0.002) respectively), although these were less prevalent than in clusters 3 and 4. This cluster had the highest proportion of individuals with moderate disease (30 %, p = 0.02). Although cluster 2 had a higher proportion of individuals with severe disease than the less inflamed cluster (27 % vs. 8.6 %, p = 0.004), mortality (3 % of tissue repair cluster 2) was comparable to the less inflamed cluster (4 %, p = 1).

The third cluster (n = 77 (25 %)) was sampled a median (IQR) of 6 (4–8) days from symptom onset. This cluster had higher levels of inflammation compared to the first two clusters, particularly markers of innate immune activation (e.g., GDF-15, GM-CSF, IL-6, and IL-15, all p < 0.001) and of Th1 and Th2 cell activation (e.g., TNF⍺, IL-33, both p < 0.001). However, the markers that were highest in this cluster were the type 1 and 2 interferons IFN⍺2a, IFNβ and IFNɣ, alveolar epithelial injury markers ST2 and RAGE and macrophage recruiting cytokine CXCL10 (all p < 0.001, except IFNβ p = 0.025). Interestingly, this cluster had a relative downregulation of markers of tissue repair, in particular EGF, PDGF-AA, PDGF-AB, VEGF and TGF⍺, but also the type 3 interferons IL28A and IL28B, endothelial activation markers e-selectin and *p*-selectin and platelet activation markers TPO and CD40L (all p < 0.001). This cluster, which we labelled a “tissue injury” cluster, composed individuals who were older (median (IQR) age 73 (60–83) years, p < 0.001), and had more individuals with immunosuppression (10 %, p = 0.009) than the other clusters, and had the highest proportion of individuals who subsequently developed severe disease (47 %, p < 0.001) and death (25 %, p < 0.001).

The fourth cluster (n = 61 (19 %)), a “systemic inflammation” cluster, was sampled a median (IQR) of 6 (3–8) days from symptom onset. This cluster was characterised by upregulation of markers encompassing all pathways, in particular innate immune activation (e.g. IL-8, procalcitonin, CCL2, TNFR1, IL-6, IL-18, GM-CSF, all p < 0.001 except CCL2 p = 0.024), immune regulation (e.g. IL1RA, PD-L1, TNF-R2, all p < 0.001), coagulation (e.g. TPO, d-dimer, both p < 0.001), and tissue repair (e.g. TGF⍺ and VEGF, both p < 0.001), but also endothelial activation (e.g. e-selectin, *p*-selectin, VCAM-1, all p < 0.001), microbial translocation (e.g. β-D-glucan, I-FABP, both p < 0.001), and axonal injury (S100B, p < 0.001). This cluster had the highest proportion of participants with diabetes (28 %, p = 0.002). Like cluster 3, this cluster was older (median (IQR) age 72 (62–81) years, p < 0.001) but had a higher BMI (median (IQR) 30 (26–33) km/m^2,^ p = 0.001). This cluster had the second highest proportion of individuals who subsequently developed severe disease (39 %, p < 0.001) or death (18 %, p < 0.001).

### 3.2 Association of inflammatory phenotypes with maximal disease severity

**Fig. 2:**
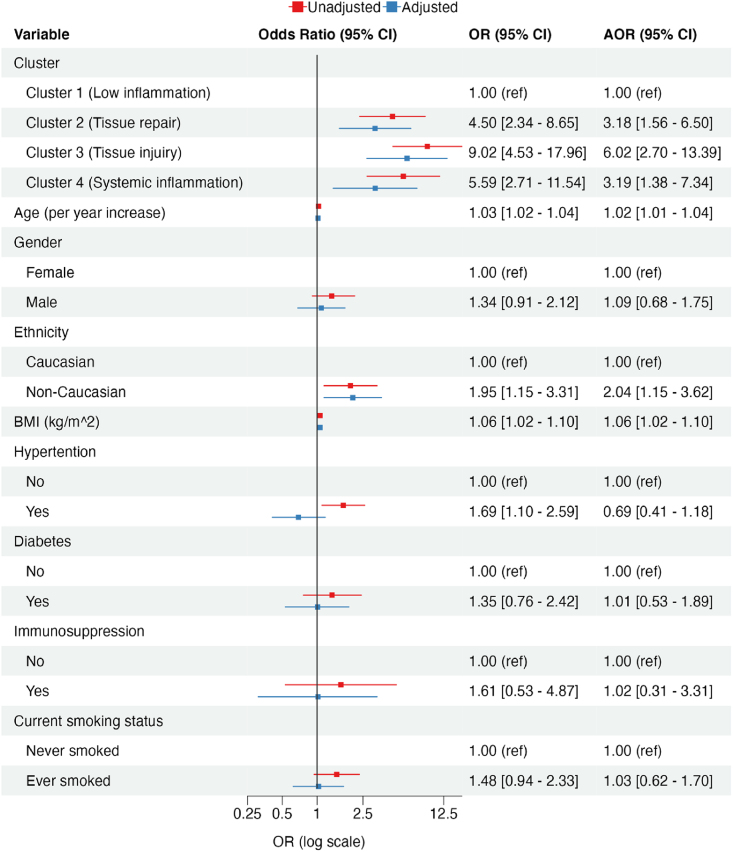
-Forest plot demonstrating unadjusted and adjusted odds of progressing to more severe COVID-19. Legend: Adjusted for age, male sex, non-Caucasian ethnicity, body mass index (BMI), hypertension, diabetes, immunosuppression, and smoking history.

As baseline demographic variables differed between groups, we next constructed ordinal logistic regression models to explore the association between cluster membership and maximal disease severity (Fig. 2). In univariable analysis, membership in clusters 2 (tissue repair), 3 (tissue injury) and 4 (systemic inflammation) were all associated with a higher odds of more severe disease compared to cluster 1 (less inflamed) [tissue repair OR (95 % CI) 4.5 (2.34–8.65), tissue injury OR (95 % CI) 9.02 (4.53–17.96), systemic inflammation OR (95 % CI) 5.59 (2.71–11.54)]. After adjustment for known clinical risk factors for severe COVID-19, including age, sex, BMI, hypertension, diabetes, immunosuppression and smoking history, clusters 2, 3 and 4 remained independently associated with a higher odds of progressing to more severe COVID-19 [tissue repair adjusted OR (aOR) (95 % CI) 3.18 (1.56–6.50), tissue injury aOR (95 % CI) 6.02 (2.70–13.39), systemic inflammation aOR (95 % CI) 3.19 (1.38–7.34)].

We constructed an additional model that was further adjusted for therapeutic steroid administration. While the addition of this variable abrogated the association between cluster 2 (tissue repair) and 4 (systemic inflammation) and disease severity [tissue repair aOR (95 % CI) 1.67 (0.75–3.74), systemic inflammation OR (95 % CI) 2.08 (0.81–5.31)], cluster 3 (tissue injury) remained significantly associated with disease severity [aOR (95 % CI) 2.45 (1.01–5.96)]. However, the dependence of steroid administration on disease severity limits the overall interpretation of this model.

## 4 Discussion

In this study, we describe four distinct inflammatory clusters present in early COVID-19, that predict varying risk of developing subsequent severe disease, independent of established risk factors for severe COVID-19. These distinct phenotypes provide new insights into the heterogeneous inflammatory pathways present in acute COVID-19. This offers the potential to advance precision medicine by targeting therapies at specific inflammatory phenotypes.

The first cluster, a low inflammation cluster, is likely representative of the majority of those with COVID-19. It comprised younger people with lower BMI, relatively few clinical COVID-19 risk factors and fewer severe cases. An early adaptive immune response that limits infection at the local tissue level may be responsible for the control of SARS-CoV-2 infection without the development a significant systemic inflammatory response [37]. These individuals may not require any specific treatment.

Cluster 2, the “tissue repair” cluster, was older, and had more clinical risk factors for severe COVID-19 but low mortality. It demonstrated upregulation of growth markers, markers of platelet and endothelial activation, but low levels of markers of early alveolar epithelial injury innate immune, Th1 and Th2 activation. Overall evidence regarding growth factors in COVID-19 is limited. Another smaller study (n = 113) that featured only individuals with moderate and severe COVID-19, found a similar enrichment of VEGF, EGF and PDGF in those with moderate disease and low mortality, when measured within an early subset (<12 days from symptom onset). One other small study demonstrated elevated VEGF and PDGF in moderate compared to severe COVID-19 [38]. Evidence for these cytokines in other pulmonary conditions is mixed, but VEGF may play a protective role in resolution from lung injury [39], and EGF is required for the repair of alveolar epithelial cells [40,41]. The profile of this cluster is consistent with an appropriate systemic inflammatory response and a proportionate regulatory response, with upregulation of growth factors [42] that facilitates resolution of lung injury. Currently available immunomodulatory therapies may be sufficient for this cluster; however further investigation of this group could provide insights into mechanisms of inflammation resolution.

In contrast, cluster 3, the tissue injury cluster, is notable for the lack of upregulation of these growth factors, type 3 interferons, platelet and endothelial markers, but high levels of markers of early alveolar epithelial injury (RAGE, ST2). This cluster had the highest prevalence of severe disease and highest mortality, with almost twice the odds of progressing to more severe disease than clusters 2 and 4 in adjusted analysis, and may represent a dysregulated inflammatory response. Early alveolar epithelial injury in COVID-19 has been demonstrated previously [43], and may contribute to the development of acute respiratory distress syndrome through the release of damage associated molecular patterns (DAMPs) [44]. This cluster had the highest levels of type 1 and 2 interferons but downregulated type 3 interferons (IL28A, IL28B). Interferon signalling has been extensively implicated in COVID-19 [45], with early balanced interferon responses thought to be central to host viral control, and delayed or dysregulated type 1 responses contributing to the cytokine storm typical of severe COVID-19. Type 3 interferons in particular have been linked to more effective immune responses and mild COVID-19 [46], while type 1 signalling promotes monocyte-macrophage driven inflammation [47]. Additionally, interferons have antiproliferative effects [48] and interfere with lung epithelial recovery following viral infection [49]. Taken together, these findings suggest that cluster 3 exhibits characteristics of dysregulated interferon responses detrimental to epithelial regeneration and tissue repair, which may explain the higher prevalence of severe disease and higher mortality. This phenotype could be a particular target for new therapies not yet widely used in COVID-19. For example, mesenchymal stromal cells, which have anti-inflammatory and tissue reparative properties, have been demonstrated to be safe in COVID-19, but efficacy is not established [50,51].

Finally, the systemic inflammatory response cluster 4 may represent individuals who subsequently develop the typical cytokine storm type observed in severe COVID-19. This cluster shares many clinical characteristics with cluster 3, but with a higher proportion of diabetes. Many factors may contribute to excessive inflammation in COVID-19, including afucosylated and inflammatory antibodies [52,53], microbial translocation [54] and increased circulation of immature myeloid cells [55]. However, immunomodulatory cytokines (IL1-RA, PD-L1, IL-10) were also increased in this cluster. For some, this counterregulatory response may have limited overall severity and prevented subsequent mortality. Individuals with this phenotype may derive benefit from earlier or more intense immunomodulatory treatment strategies. For example, the use of higher dose corticosteroids, which has had conflicting results in studies to date [56,57], may provide benefit if administration is focused on individuals with this inflammatory pattern.

There have been previous attempts to identify immunophenotypes in acute COVID-19, most of which examine the risk of severe disease or death with individual biomarkers. Many of these studies are limited by a small sample size [[58], [59], [60]], include individuals later in the disease course [13] or lack detail on time from symptom onset altogether [61,62]. Three previous studies have used unsupervised clustering on cytokines in acute COVID, all of which included individuals later in the disease course, and two of which [63,64] were substantially smaller than this analysis (n = 138 and n = 113, respectively). While the third study [65] was larger (n = 471), a more limited panel of cytokines was examined (n = 33), and time from symptom onset was significantly later in those with more severe disease, which prevented detection of early changes that precede clinical deterioration. While comparison of clusters with these studies is limited due to differences in study design, all found a less inflamed cluster and a more highly inflamed cluster, like the low inflammation and systemic inflammatory response patterns seen here. One other study [64] had a pattern similar to the tissue repair cluster with upregulation of growth factors and less severe outcomes. None of these studies explored the association between inflammatory phenotype and disease severity, controlling for clinical factors. The present study is large and measured a large panel of cytokines covering the major inflammatory pathways implicated in COVID-19 which allowed a detailed immunophenotyping of individuals early in the disease course, prior to clinical deterioration, during which intervention could occur. The availability of clinical data in this cohort allowed us to identify an inflammatory phenotype, the tissue injury cluster 3, associated with the most severe disease, that cannot be explained by clinical risk factors. Our findings offer novel insights that could advance individualised therapy.

This analysis has limitations. Recruitment occurred early in the pandemic, during wild-type and alpha-dominant waves, restricting assessment of potential changes in phenotype with more recent variants. However, this was before widespread reinfection and vaccination, allowing the study of inflammatory changes in susceptible hosts. Although we focused on the early disease course when intervention is most likely to be effective, we performed only a cross sectional assessment of immunophenotype, precluding an evaluation of immune trajectories over time. We examined only circulating plasma-based markers, and not cell based or functional markers in this study, which have been implicated in the pathology of COVID-19 [[66], [67]], but unlike soluble biomarkers, cellular markers are not widely used in routine clinical labs, limiting their potential as predictive markers. We did not incorporate biomarkers from routine lab data, such as neutrophil to lymphocyte ratio, which have demonstrated prognostic value [68,69]. While this study was multi-centre, it was based in a single country, and further study is needed to validate these findings in other populations. We did not assess the impact of inflammatory phenotype on post-acute sequelae or incidence of long COVID, which carries substantial morbidity and remains poorly understood [[70], [71], [72]].

In summary, in this study, we defined unique host immune profiles in early COVID-19, with distinct inflammatory pathways resulting in greater disease severity. Future studies should focus on identifying a smaller set of markers that best identify an inflammatory cluster and validation in longitudinal cohorts examining clinical outcomes following severe acute viral infection in susceptible hosts. This would allow the development of inflammatory phenotype guided clinical trials to improve therapeutic strategies in COVID-19 and future viral respiratory infections.

## Funding

This analysis was supported by Science Foundation Ireland (grant numbers 20/COV/8549 and SFI 20/COV/8566) and philanthropic donation from Smurfit Kappa. GK was funded through a fellowship from the United States Embassy in Ireland during this study.

## 5 Data availability statement

Data associated with this manuscript can be requested from the All Ireland Infectious Diseases Cohort Study group, and will be made available on request subject to approval by a local ethics committee.

## CRediT authorship contribution statement

**Grace Kenny:** Writing – original draft, Investigation, Formal analysis, Data curation, Conceptualization. **Gurvin Saini:** Writing – review & editing, Investigation. **Colette Marie Gaillard:** Writing – review & editing, Investigation. **Riya Negi:** Writing – review & editing, Investigation. **Dana Alalwan:** Writing – review & editing, Investigation. **Alejandro Garcia Leon:** Writing – review & editing, Supervision, Investigation. **Kathleen McCann:** Writing – review & editing, Data curation. **Willard Tinago:** Writing – review & editing, Supervision, Formal analysis. **Christine Kelly:** Writing – review & editing, Supervision, Data curation, Conceptualization. **Aoife G. Cotter:** Writing – review & editing, Project administration. **Eoghan de Barra:** Writing – review & editing, Project administration. **Mary Horgan:** Writing – review & editing, Project administration. **Obada Yousif:** Writing – review & editing, Project administration. **Virginie Gautier:** Writing – review & editing, Supervision. **Alan Landay:** Writing – review & editing, Supervision, Conceptualization. **Danny McAuley:** Writing – review & editing, Project administration, Funding acquisition, Conceptualization. **Eoin R. Feeney:** Writing – review & editing, Project administration, Investigation, Funding acquisition, Conceptualization. **Cecilia O'Kane:** Writing – review & editing, Resources, Project administration, Investigation, Data curation, Conceptualization. **Patrick WG. Mallon:** Writing – review & editing, Supervision, Project administration, Investigation, Funding acquisition, Formal analysis, Conceptualization.

## Declaration of competing interest

The authors declare the following financial interests/personal relationships which may be considered as potential competing interests: Patrick Mallon reports financial support was provided by 10.13039/501100001602Science Foundation Ireland. Patrick Mallon reports financial support was provided by Smurfit Kappa Group plc. Grace Kenny reports financial support was provided by United States Embassy in Ireland. Eoin Feeney, Paddy Mallon and Alan Landay reports a relationship with Gilead Sciences Inc that includes: board membership, consulting or advisory, and travel reimbursement. Eoin Feeney and Paddy Mallon reports a relationship with ViiV Healthcare that includes: consulting or advisory and travel reimbursement. Eoin Feeney reports a relationship with Vidacare Ireland that includes: consulting or advisory. Eoghan de Barra reports a relationship with Sanofi Pasteur Inc that includes: consulting or advisory. Eoghan de Barra reports a relationship with Pfizer Inc that includes: travel reimbursement. Paddy Mallon reports a relationship with MSD that includes: consulting or advisory and travel reimbursement. Paddy Mallon reports a relationship with AstraZeneca Pharmaceuticals LP that includes: consulting or advisory and travel reimbursement. Alan Landay reports a relationship with Abbott Laboratories that includes: consulting or advisory. Cecilia O'Kane reports a relationship with Insmed Inc that includes: consulting or advisory. Cecilia O'Kane reports a relationship with California Institute of Regenerative Medicine that includes: board membership. Cecilia O'Kane and Danny McCauley reports a relationship with 10.13039/100018645MRC that includes: funding grants. Cecilia O'Kane and Danny McCauley reports a relationship with 10.13039/100010269Wellcome Trust that includes: funding grants. Cecilia O'Kane and Danny McCauley reports a relationship with National Institute of Health Sciences that includes: funding grants. Cecilia O'Kane and Danny McCauley reports a relationship with 10.13039/501100006041Innovate UK that includes: funding grants. Danny McCauley reports a relationship with 10.13039/100014101Novavax Inc that includes: funding grants. Danny McCauley reports a relationship with 10.13039/100004330GlaxoSmithKline that includes: consulting or advisory and speaking and lecture fees. Danny McCauley reports a relationship with Bayer that includes: consulting or advisory. Danny McAuley reports a relationship with Boehringer Ingelheim that includes: consulting or advisory. Danny McAuley reports a relationship with Novartis that includes: consulting or advisory. Danny McAuley reports a relationship with Eli Lilly and Company that includes: consulting or advisory. If there are other authors, they declare that they have no known competing financial interests or personal relationships that could have appeared to influence the work reported in this paper.
