# Supplementary material for: Early inflammatory profiles predict maximal disease severity in COVID-19: An unsupervised cluster analysis
Source: Heliyon. 2024 Jul 23;10(15):e34694. doi: 10.1016/j.heliyon.2024.e34694 (PMC11320140; doi:10.1016/j.heliyon.2024.e34694)
Supplement: Multimedia component 1 [file mmc1.docx]

**Supplemental Information**

Supplementary table 1 - Biomarker panels

|  | Markers measured |
| --- | --- |
| Panel 1 – Luminex platform – 22 markers | IL-28A/IFN-lambda 2, PD-L1/B7-H1, IL-8/CXCL8, CXCL10/IP-10/CRG-2, S100B, EGF, VEGF, CD163, IL-1ra/IL-1F3, CCL3/MIP-1 alpha, D-dimer, E-Selectin/CD62E, GM-CSF, P-Selectin/CD62P, Thrombopoietin/Tpo, PDGF-AA, ICAM-1/CD54, CCL2/JE/MCP-1, TGF-alpha, IL-28B/IFN-lambda 3, CD40 Ligand/TNFSF5, IL-18/IL-1F4 |
| Panel 2 – Luminex platform – 10 markers | IL-33, FABP4/A-FABP, CCL22/MDC, Procalcitonin, TSLP, Resistin, Leptin/OB, IL-15, TNF RII/TNFRSF1B, TNF R1/TNFRSF1A |
| Panel 3 – Luminex platform – 5 markers | CD14, Serpin E1/PAI-1, Adiponectin/Acrp30, LBP, C-Reactive Protein/CRP |
| Panel 4 - Luminex platform – 3 markers | vWF-A2, PDGF-AB, VCAM-1 |
| Panel 5 – MSD platform – 10 markers | IL-10, IL-12p70, IL-13, IL-17A, IL-1beta, IL-2, IL-4, IL-5, IL-6, TNF-alpha |
| Panel 6 – MSD platform – 4 markers | IFN-Alpha1a, IFN-Beta, IFN-Gamma, IFN-Lambda 1 |
| Markers run in single plex | I-FABP, Zonulin, Beta-D-Glucan, GDF-15, ST2, SPD RAGE |

Supplementary figure 1 – Scree plot from the Prinicipal Component Analysis (PCA), demonstrating the variance explained by the first 10 dimensions

Supplementary Figure 2 – Dendrogram of the agglomerative hierarchical cluster analysis.
